# Structural determinants influencing halogen bonding: a case study on azinesulfonamide analogs of aripiprazole as 5-HT_1A_, 5-HT_7_, and D_2_ receptor ligands

**DOI:** 10.1186/s13065-018-0422-5

**Published:** 2018-05-11

**Authors:** Krzysztof Marciniec, Rafał Kurczab, Maria Książek, Ewa Bębenek, Elwira Chrobak, Grzegorz Satała, Andrzej J. Bojarski, Joachim Kusz, Paweł Zajdel

**Affiliations:** 1 Department of Organic Chemistry, Medical University of Silesia, 4 Jagiellońska Street, 41-200 Sosnowiec, Poland; 2 Department of Medicinal Chemistry, Institute of Pharmacology, Polish Academy of Sciences, 12 Smętna Street, 31-343 Krakow, Poland; 3 Institute of Physics, University of Silesia, 4 Uniwersytecka Street, 40-007 Katowice, Poland; 4 Department of Medicinal Chemistry, Jagiellonian University Medical College, 9 Medyczna Street, 30-688 Krakow, Poland

**Keywords:** Azinesulfonamides, Long-chain arylpiperazine, Aripiprazole, Crystal structure, Halogen bond

## Abstract

A series of azinesulfonamide derivatives of long-chain arylpiperazines with variable-length alkylene spacers between sulfonamide and 4-arylpiperazine moiety is designed, synthesized, and biologically evaluated. In vitro methods are used to determine their affinity for serotonin 5-HT_1A_, 5-HT_6_, 5-HT_7_, and dopamine D_2_ receptors. X-ray analysis, two-dimensional NMR conformational studies, and docking into the 5-HT_1A_ and 5-HT_7_ receptor models are then conducted to investigate the conformational preferences of selected serotonin receptor ligands in different environments. The bent conformation of tetramethylene derivatives is found in a solid state, in dimethyl sulfoxide, and as a global energy minimum during conformational analysis in a simulated water environment. Furthermore, ligand geometry in top-scored complexes is also bent, with one torsion angle in the spacer (τ_2_) in synclinal conformation. Molecular docking studies indicate the role of halogen bonding in complexes of the most potent ligands and target receptors.

**Electronic supplementary material:**

The online version of this article (10.1186/s13065-018-0422-5) contains supplementary material, which is available to authorized users.

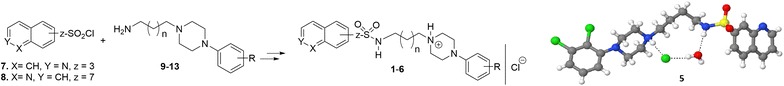

## Introduction

Long-chain arylpiperazines (LCAPs) constitute one of the largest classes of serotonin (5-HT), and dopamine (D) receptor ligands, and exhibit diverse actions on the central nervous system (CNS) [1–4]. Among this vast group, we recently developed LCAP analogs of aripiprazole, with quinoline- or isoquinoline-sulfamoyl moieties, which displayed a 5-HT/D multi-receptor binding profile [5–8]. Structure–activity relationship studies have revealed that the observed receptor binding and functional profiles depend on the type of substituent in the arylpiperazine moiety, and the length and conformation of the aliphatic linker and the terminal fragment. Completing the characterization of this class of ligands, the selected compounds show potent antidepressant or antipsychotic activity with pro-cognitive properties [5–8].

**Fig. 1:**
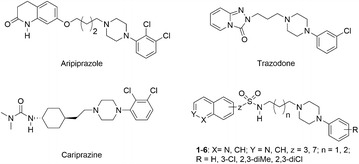
Chemical structures of atypical antipsychotics aripiprazole, trazodone, cariprazine and compounds used in this study

In recent years, many research groups have explored monochloro- or dichloro-phenylpiperazine as a privileged structure for the optimization of CNS-active compounds providing with such psychotropic drugs as aripiprazole, trazodone, cariprazine [9–12] (Fig. 1). In the following years, the first reports were published on the engagement of halogen atoms in stabilization of the ligand–receptor complex within compounds targeting the central nervous system, especially 5-HT_1A_, 5-HT_7_, and D_2_ receptors [13].

Some controversy has arisen concerning the role of the alkylene linker, usually composed of two to five carbon atoms as to whether it actively participates in binding or simply acts as a distance arm providing a chain [14]. Nevertheless, due to the highly flexible nature of a linker, various attempts have been made to determine the bioactive conformation of LCAPs.

Assuming that active conformations of LCAPs are closely related to those in solution or in solid state, two-dimensional nuclear magnetic resonance (2D NMR) and crystallographic methods have often been applied to approximate the bioactive structure [15–20]. These 2D NMR studies indicate that compounds with an alkylene spacer can adopt extended, bent, or folded conformations [15–17]. In contrast, analysis of the Cambridge Structural Database (CSD) indicates that linear geometries are predominant (see Additional file 1: Table S1). Furthermore, molecular modeling simulations (conformational analysis and docking experiments) have provided equivocal results on the different bioactive conformations of LCAPs.

Extending studies on verification of the impact of halogen bond, alkylene linker length, and localization of the sulfonamide group in the azine moiety, a limited series of isoquinoline-sulfonamide derivatives of LCAP were designed (Fig. 1). Following our previous studies [5] suggesting the preferential position of the sulfonamide group in the β-position of the azinyl moiety, regardless of sulfonamide group localization in pyridine or benzene rings, the 3-isoquinolyl moiety was selected for the design of new aripiprazole analogs. Herein we report on the synthesis of selected azinesulfonamides and their X-ray structure analysis, followed by NMR experiments, and in silico molecular modeling. In doing so, we attempt to understand the conformational orientation of chemical sub-structures favorable for interaction with 5-HT_1A_ and 5-HT_7_Rs.

## Results and discussion

### Source of compounds

**Scheme 1:**
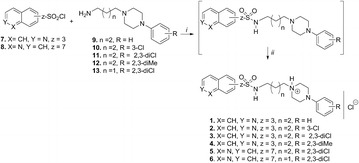
Synthesis of azinesulfonamides **1**–**6**. Reagents and conditions: (i) DIEA, CH_2_Cl_2_, 0 °C/rt.; (ii) 1 M HCl in dioxane/rt

Azinesulfonamide analogs of aripiprazole **1**–**6** were prepared according to previously reported procedures (Scheme 1) [5, 6].

The synthesis of compounds **1**–**6** was carried out by reaction of the primary amines (**9**–**13**) with 3-isoquinolinesulfonyl chloride (**7**) or 7-quinolinesulfonyl chloride (**8**) in the presence of Hunig’s base. The azinesulfonyl chlorides **7** and **8** were prepared from 3-bromoisoquinoline or 7-chloroquinoline, respectively, according to the previously reported method [5]. For the pharmacological evaluation, free bases were converted into their water-soluble hydrochloride salts **1**–**6**. The spectroscopic data (NMR and MS) of compounds **3**, **5**, and **6** were identical to those previously reported [5].

### Structure–activity relationship studies

Following our previous studies [5], which suggest a preferential position of sulfonamide group in the β-position of the azinyl moiety (confirming the structural analogy for the dihydroquinolin-7-yl-2-one core in aripiprazole), 3-isoquinolyl moiety was selected to design the analogs of aripiprazole. In a series of new isoquinolinyl derivatives, we also focused our attention on the type of halogen substitution in a phenylpiperazine fragment to determine the role of the halogen bond in ligand complexes and target receptors.

The term “halogen bond” refers to the non-covalent interactions of halogen atoms X in one molecule with a negative site on another. X can be chlorine, bromine or iodine, but not fluorine. It is increasingly recognized that halogen bonding occurs in various biological systems and processes, and can be utilized effectively in drug design [21, 22]. Subsequently, regarding preliminary studies on the engagement of halogens in the interaction of LCAP derivatives with a partially rigidified alkylene spacer with serotonin receptors [8], our interest was focused on the impact of halogen in binding of compounds **2**–**6** with 5-HT_1A_ and 5-HT_7_ receptors.

The unsubstituted analog **1** displayed low affinity for all tested receptors (Table 1). Introduction of chlorine in the 3-position increased receptors’ affinity up to 3–12-fold (**1** vs **2**). These findings are in line with our previous study, and reveal that the presence of a chlorine atom in the 3-position stabilizes the ligand–receptor complex through the formation of a halogen bond with Thr5.39 residue of 5-HT_1A_ and 5-HT_7_Rs [8]. Furthermore, introduction of a second chlorine atom in the 2-position of the phenylpiperazine yielded compound **3**. This modification did not substantially affect the receptor binding profile in comparison to its 3-chloro counterpart (**2** vs **3**), except for an increase in the affinity for D_2_Rs. In contrast, replacement of chlorine atoms in the 2- and 3-positions with methyl groups (compound **4**) decreased the affinity for 5-HT_1A_, and 5-HT_7_Rs up to twofold (**3** vs **4**).

**Table 1:** Binding affinity of the investigated azinesulfonamides **1**–**6** for 5-HT_1A_, 5-HT_6_, 5-HT_7_, and D_2_ receptors

Compound				K_i_ (nM)^a^			
	Azinyl	n	R	5-HT_1A_	5-HT_6_	5-HT_7_	D_2_
**1**	3-isoquinolinyl	2	H	304	1352	245	565
**2**	3-isoquinolinyl	2	3-Cl	38	436	49	47
**3** ^b^	3-isoquinolinyl	2	2,3-diCl	34	454	56	17
**4**	3-isoquinolinyl	2	2,3-diMe	73	916	85	23
**5** ^b^	7-quinolinyl	2	2,3-diCl	17	301	31	11
**6** ^b^	7-quinolinyl	1	2,3-diCl	14	257	12	16
**Aripiprazole**		–	–	5.6	90	26	0.8

^a^ Mean *K*_i_ values (SEM ± 23%) based on three independent binding experiments

^b^ Data taken from Ref. [6]

Subsequently, we compared the data obtained for 3-chloro- and 2,3-dimethyl derivatives (**2** and **4**, respectively) and unsubstituted phenylpiperazine analog **1** with those previously reported for their 2,3-dichloro analogs. A change of the 3-isoquinolinyl fragment for 7-quinolinyl yielded compound **5**, which displayed a two- to three-fold higher affinity for 5-HT_1A_, 5-HT_6_, and 5-HT_7_Rs, thus revealing the 7-quinolinyl fragment as more favorable for interaction with 5-HT_1A_Rs. Within the evaluated quinoline derivatives **5** and **6**, shortening of the butylene spacer to propylene one, had little influence on the receptor profile.

The binding data for D_2_ receptors revealed that compound **1**, unsubstituted at the phenyl ring, displayed low affinity for D_2_Rs with *K*_i_ equaling 565 nM (Table 1). Introduction of one chlorine atom in the 3-position increased affinity for D_2_Rs 12-fold, and two chlorine atoms in the 2- and 3-positions increased affinity up to 33-fold. As found in our previous research [8], replacement of chlorine atoms with methyl substituents maintained affinity for D_2_Rs at the same level. This could suggest a lower impact of halogen bonds, as they are less engaged in interaction with D_2_Rs then in the case of 5-HT_1A_ and 5-HT_7_Rs. Furthermore, it was found, that the 3-isoquinolinyl fragment was less preferable than 7-quinolinyl for interaction with D_2_Rs (**4** vs **5**) and shortening of the alkylene linker (from 4 to 3 methylene units) maintained a high affinity for D_2_Rs (**4** vs **6**).

Generally, the compounds selected for extended structural evaluation may be classified as multimodal serotonin and dopamine receptor ligands with high affinity for 5-HT_1A_/5-HT_7_ and D_2_ receptors, and moderate to low affinity for 5-HT_6_ receptors. Significantly, introduction of an azinesulfonamide group into the structure of LCAPs, decreased their affinity for D_2_ receptors compared to aripiprazole [8].

### Structural analysis

The Cambridge Structural Database (CSD version 5.39, November 2017 [23]) was used to search for compounds with the following queries: unsubstituted piperazine carbon atoms, no additional cyclic arrangements between aryl and piperazine moieties with ethylene, propylene, and butylene spacers. The search resulted in 36 hits (Additional file 1: Table S2). The piperazine ring in all structures deposited in the CSD adopts the chair conformation with substituents located equatorially. The mutual position of aryl and piperazine rings may be described simply by the torsional angle τ and/or dihedral angle ф between piperazine plain and the phenyl ring (Additional file 1: Figure S1). In the majority of *meta*- and *para*- substituted derivatives, the phenyl ring is more or less coplanar with piperazine (torsion angle values are grouped in the vicinity of 0° or 180°, while the dihedral angle is far from 90°). At the same time, all *ortho*-substituted compounds exhibit noncoplanar conformation. The spacers’ conformations vary from fully extended to variously bent. Methylene chains predominantly adopt the extended form in the crystal. Meanwhile, in arylpiperazine salts piperazine nitrogen N1 is protonated and interacts with the respective anion through inter-ionic hydrogen bonds. These interactions establish a salt bridge between the molecules, which plays a leading role in the discussed crystal architecture. In the case of solvent-free hydrochlorides, NH^+^···Cl^−^···H–C_spacer_ interactions form a simple short bridge between more or less parallel molecules. Salt bridge elongation was observed in a number of structures containing water in the form of NH^+^···Cl^−^···H_2_O···H–C_spacer_. Furthermore, these interactions caused important variation in the conformation of the alkylene spacer [20, 24].

**Fig. 2:**
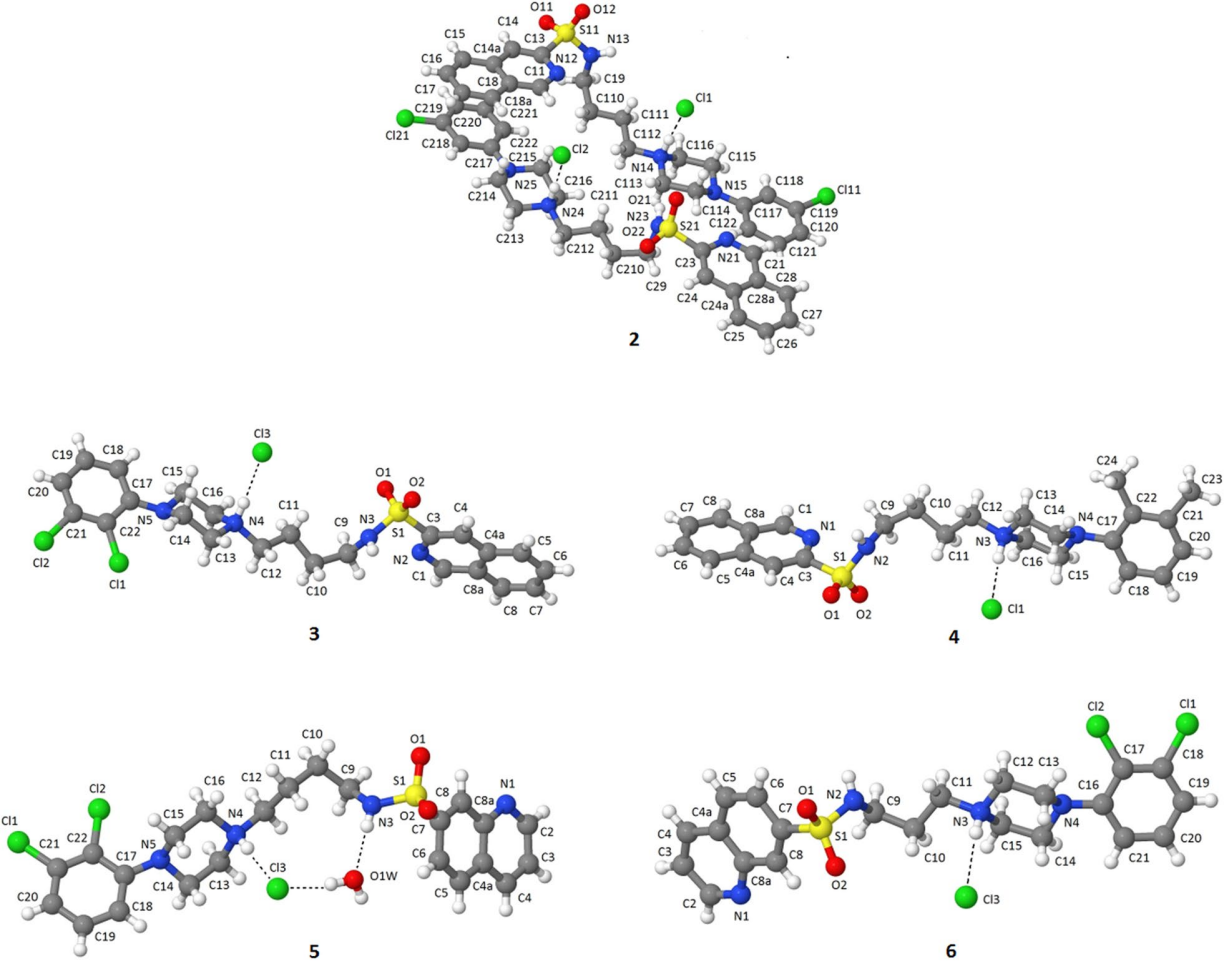
Molecular geometry of crystal structures, showing the atom labelling scheme. Dashed lines represent a charge-assisted hydrogen bond NH^+^···Cl^−^ or NH^+^···Cl^−^···H_2_O···H–N_sulfon_

Most of the above observations concerning the conformations of arylpiperazines collected in the CSD are self-evident for the five structures investigated in this paper. The main difference in the crystal structures of compounds **2**–**6** is the construction of salt bridges. Introduction of the –SO_2_–NH– sulfonamide fragment provides two strong proton acceptors and one strong proton donor which significantly change inter-ionic interaction in azinesulfonamides **2**–**6** compared to the crystal structures deposited in the CSD. In the case of solvent-free hydrochlorides, the NH^+^···Cl^−^···H–N_sulfon_ interactions form a simple short bridge. Salt bridge elongation, resulting from water participation, was observed in one structure in the form of NH^+^···Cl^−^···H_2_O···H–N_sulfon_ in **5** (Fig. 2).

It should be mentioned that all derivatives **1**–**6** are highly resistant to crystallization and dissolve in most solvents; the glassy state is a dominant solid state for these derivatives. Therefore, we were fortunate to successfully obtain the monocrystals for five analogs of LCAPs. It should be pointed out that studying the structure nonsubstituted at phenyl ring analog **1** was also planned; however, owing to the crystal quality only a rough structure model was obtained.

In the structure of **2**, two molecules were found in an independent unit, with the conformation of both molecules being almost identical. Moreover, in the structure of hydrochloride **5**, besides the chlorine anion, a water molecule was also found. Significant geometrical parameters of the studied structures are summarized in Table 2.

**Table 2:** Selected interatomic distances [Å] and dihedral angles [º]^a^ of the studied compounds

Compound	*N*^+^···*N*_sulfon_	τ_1_	τ_2_	τ_3_	τ_4_	τ_5_	τ_6_	Ф
**2**	5.47	− 113.7	− 69.0	179.4	− 174.8	53.7	− 147.5	20.6
**3**	5.48	− 156.3	74.4	− 170.5	− 176.5	− 49.5	159.2	47.1
**4**	5.48	− 155.7	73.9	− 171.4	− 176.2	− 50.5	158.8	48.0
**5**	5.00	− 119.3	− 65.1	− 72.7	178.3	− 53.7	− 143.8	62.8
**6**	4.39	− 102.7	− 68.3	179.7	− 55.1	–	154.6	51.5

^a^ For the definition of dihedral angles see Fig. 3

In the crystal structures of compounds **2**–**6** the piperazine ring was in a common chair conformation (with the two N-substituents in equatorial positions) as indicated by deviations of nitrogen atoms in opposite directions from the plane defined by the ring carbons. The resulting distances were 0.63 and 0.64 Å for **2**, 0.73 and 0.64 Å for **3**, 0.73 and 0.64 Å for **4**, 0.72 and 0.62 Å for **5**, and 0.72 and 0.62 Å for **6**, respectively, with the second values referring to the protonated piperazine nitrogen, substituted equatorially by the alkylene linker. Accordingly, the inter-correlated position of piperazine and aromatic rings of the LCAPs may play a crucial role in ligand receptor recognition. The arylpiperazine moiety in the 2,3-disubstituted at phenyl ring azinesulfonamides **3**–**5** exhibits non-coplanar conformation with the main piperazine plane (formed by the atoms C13, C14, C15, and C16) and the phenyl inclined by ф = 47.1–62.8°. In the 3-substituted derivative **2**, the phenyl ring is more coplanar with the piperazine plane (ф = 20.6°) (Table 2). It is worth mentioning that in sulfonamides **5** and **6**, the most potent 5-HT_1A_ and 5-HT_7_ receptor ligands, the angle between the piperazine plane and the phenyl ring reached its highest values (51.5° and 62.8° respectively). As a result, ability of the chlorine atom to stabilize the ligand–receptor (L–R) complex by the formation of stronger halogen bonds is increased compared with compounds **2** and **3**. Furthermore, the quinolinesulfonamide heterocyclic head and phenyl ring in compounds **5** and **6** were essentially planar, while in isoquinolinesulfonamides the phenyl and isoquinoline planes were almost perpendicular to each other in the crystals of **3** and **4** (ф = 81.6° and 77.6°, respectively).

**Fig. 3:**
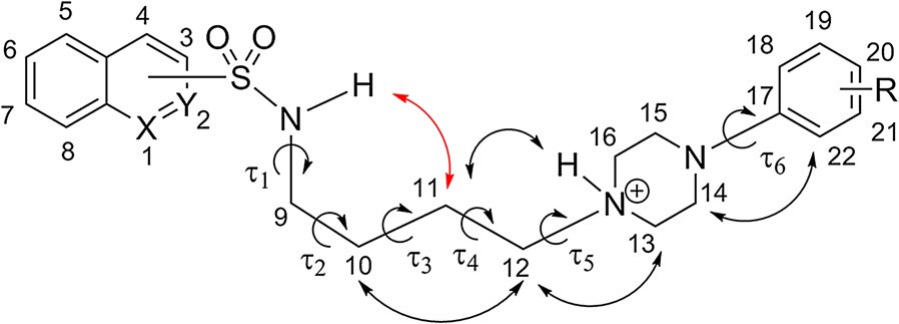
Numbering system used in X-ray and NMR analysis of azinesulfonamides with tetramethylene linker. Significant NOE signals were also demonstrated

Special attention was placed upon the conformation of the alkylene spacer, due to its significant flexibility. Analysis of similar structures found in the CSD showed that an extended conformation of the spacer was favorable (see Additional file 1: Table S1). The five new crystallographic structures obtained results that differed slightly from the protonated analogs of alkylarylpiperazines deposited in the CSD. In compound **6,** the *n*-propyl chain adopted a bent conformation gauche-trans–trans (for torsion angle see Table 2) and in the **2**–**5** conformation was not fully extended. The bending of the chain (gauche conformation) on the C9–C10 bond was essential for the obtained crystal structures (Fig. 3).

The supramolecular organization of the hydrochloride **2** was based mainly on different weak hydrogen bonds of the type C–H···Cl. Additionally one strong hydrogen bond between the nitrogen NH^+^ and Cl^−^ anion was observed. The molecules were linked by weak hydrogen bonds between carbon atoms from the phenylpiperazine rings and oxygen atoms from the sulfonamide group. Additional file 1: Table S3 contains detailed characteristics of these interactions.

The solid-state conformations of azinesulfonamides **3** and **4** are stabilized by a system of intermolecular hydrogen bonds. The geometric parameters indicate that in the crystal structure of compounds **3** and **4**, molecules of the studied sulfonamides form chains, of the head-to-tail type stabilized by salt bridges of NH^+^···Cl^−^···H–N_sulfon_ (Additional file 1: Table S3).

**Fig. 4:**
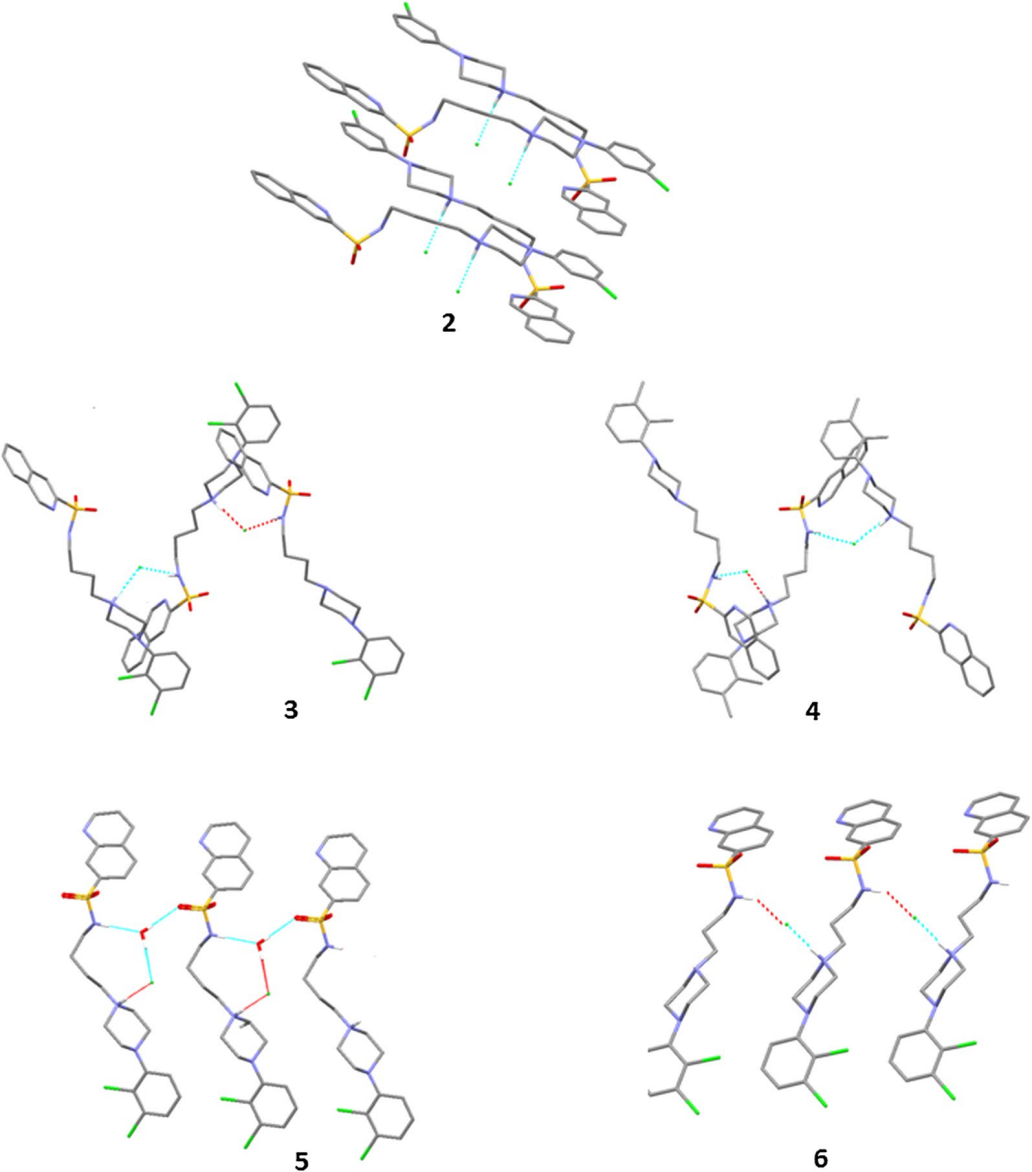
Crystal packing of azinesulfonamides **2**–**6**. Hydrogen atoms not involved in the hydrogen bond patterns were removed for clarity

Meanwhile, molecules of sulfonamide **5**, as well as sulfonamide **6**, were joined as a head-to-head type chain motif, with intermolecular distance equal to 6.91 Å. The type of interaction that governs the crystal packing of the presented structure is strong hydrogen bonds, in which water molecules are involved. The observed hydrogen bond motives differ for sulfonamide **5** (Fig. 4), and the solvent molecule creates interesting patterns in the crystal lattice. Water molecules form the strong hydrogen bonds O–H···Cl^−^···NH^+^ and H–O···H–N_sulfon_. Moreover, in the crystal structure of **5**, water is a hydrogen bond donor with the quinolinesulfonamide oxygen atom acting as an acceptor (Additional file 1: Table S3).

The supramolecular organization of hydrochloride **6** is primarily governed by strong inter-ionic hydrogen bonds between the protonated arylpiperazine nitrogen, the chlorine anion located in the gap between extended molecules and nitrogen of the sulfonamide group (Fig. 4). Thus, due to steric and geometrical complementarities, parallel molecules of **6** form chains of the head-to-head type, with an intermolecular distance equal to 6.82 Å. In this arrangement, molecules are joined by salt bridges of NH^+^···Cl^−^···H–N_sulfon_ (graph set notation of *C*_*2*_^*1*^(8)) (Additional file 1: Table S3).

### NMR studies

In the solid state, the compound exists in a bent conformation with the methylene bridging units in a synclinal–antiperiplanar–antiperiplanar–antiperiplanar arrangement for compounds **2**–**5**, and a synclinal–antiperiplanar–antiperiplanar arrangement for compound **6**. Since the chemical shift and multiplicity of two methylene groups (H-10 and H-11) in the tetramethylene bridging units can provide information about the conformational preferences of tested compounds, ^1^H NMR studies for **2**–**5** were performed. In general, the ^1^H NMR spectra of azinesulfonamides analyzed in DMSO solutions, were characterized by two multiplets separated by 0.25–0.27 ppm, assigned to the protons of two central methylene groups (H-10 and H-11) of the butyl chain. This might suggest the bent conformation, which is in agreement with previous observations [17, 25]. The above inferences were confirmed by the *nuclear Overhauser effect* (NOE). This experimental evidence for the conformations in solution of compounds **2**–**5** was provided by *rotating frame Overhauser effect spectroscopy* (ROESY) experiments conducted in DMSO; the significant NOE signals are indicated in Fig. 3. If the compounds were in their extended conformation, the interactions between the sulfonamide proton and H-11 would not be expected to exist. Alternatively, in a bent conformation the closer spatial arrangements of these protons could explain the observed NOE signals. In the obtained spectra, characteristic cross-peaks from the sulfonamide proton and methylene protons of the alkyl chain (H-11) were assigned the bent conformation. On the other hand, the appearance of weak interactions between H-9 and H-11 protons indicates the possibility of equilibrium between the bent and the extended conformation. However, the lack of interactions between the azine moiety and the phenyl protons (from arylpiperazine) definitely excludes the folded conformation of compounds and stacking interaction in solution.

Furthermore, 2D-NOE experiments confirmed the cross peaks for intramolecular interactions, are thus in agreement with bent conformations in solution. Molecular modeling studies of azinesulfonamides **2**–**6** were subsequently carried out with Gaussian 16 computer code. Conformational preferences were explored using the parameters for either the isolated “gas phase” or water continuum. Among the structures produced, the free energetically favored conformations indicated the methylene C9 and C10 bridging groups in a synclinal arrangement with aromatic portions far away from each other, consistent with NMR experimental data (Additional file 1: Table S4 and Figure S2). Higher energy extended structures were also generated, which were approximately 85 kJ/mol above the bent structures at most. This data constituted the basis for further molecular modeling and prediction of the ligands’ binding orientation to a receptor binding site.

### Molecular modeling

**Fig. 5:**
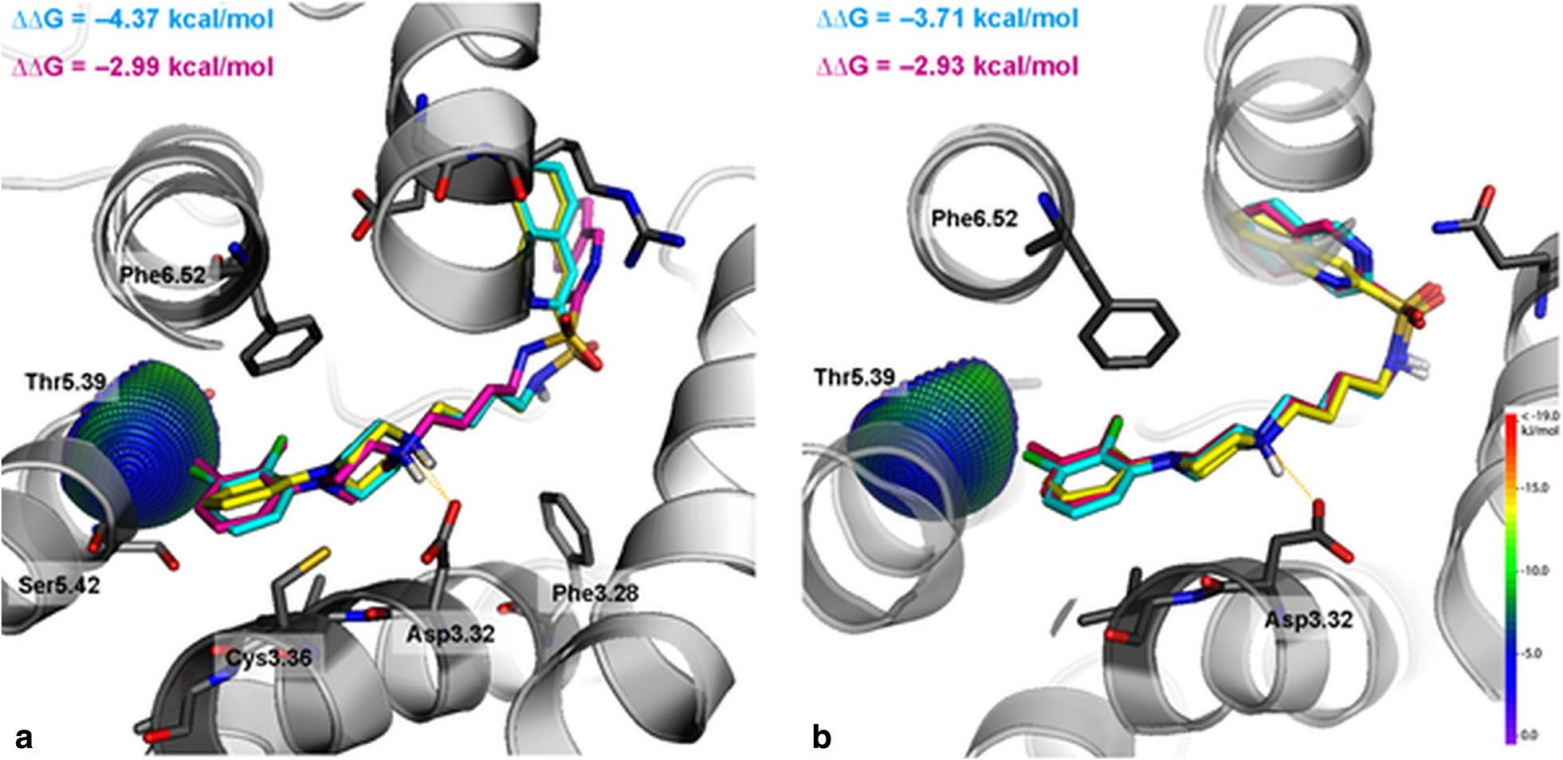
Superposition of the poses of compounds **1** (yellow), **4** (magenta), and **5** (cyan) against putative halogen binding pocket interaction spheres for 5-HT_7_ (**a**) and 5-HT_1A_ (**b**) receptors, respectively. The chlorine–oxygen theoretical interaction spheres illustrate the projected qualities of the formed ligand–receptor halogen bonds. The applied methodology is described by Wilcken et al. [33]

To complete the examination of the conformational preferences of the studied compounds, molecular docking of azinesulfonamides was performed with the use of recently developed 5-HT_7_ and 5-HT_1A_ homology models, built on a dopaminergic D_3_ receptor template (PDB ID: 3PBL) [13, 26–31]. Next, the combination of the QPLD with MM-generalized-born/surface area (MM/GBSA) calculations from the Schrödinger Suite was used to obtain ligand–receptor complexes, as this approach is suitable to describe the anisotropy of the electron density of halogen atoms, which is a key feature during halogen bond examination [32]. The obtained complexes (Fig. 5) exhibit highly consistent binding modes, involving a salt-bridge with Asp3.32 and interactions formed by the aromatic moiety of the arylpiperazine fragment (CH–π) with the side chain of Phe6.52. The higher affinity of **5** (with 2,3-dichloro substituent) for 5-HT_7_ and 5-HT_1A_ than its unsubstituted analog **1** might be explained by the ability of chlorine to stabilize the ligand–receptor complex by the formation of a halogen bond (Cl∙∙∙O distance = 3.38 Å, σ-hole angle = 177.6° for 5-HT_7_R, and Cl∙∙∙O distance = 3.62 Å, σ-hole angle = 167.9° for 5-HT_1A_R, respectively) with the backbone carbonyl group of Thr5.39 (Fig. 5).

The change in binding affinities for compound **5** is probably related to the mutual orientation of the piperazine plane and the phenyl ring. In complexes of compound **5** with receptors, the angle between both rings maintains high values (57.4° for 5-HT_1A_ and 67.3° for 5-HT_7_, respectively). Interestingly, in both receptors a higher increase in binding free energy (∆∆G) was noted for the dihalogenated (**5**) than dimethylated (**4**) analog of compound **1**, indicating a significant role of halogen bonding in ligand–receptor interaction.

Among all of the docked poses, the bent conformations predominated and no extended arrangements were found (Additional file 1: Table S4). This is in general agreement with the parameters of related crystal structures as well as with NMR experimental data.

## Conclusions

The present paper has reported the preparation and performance of biological and conformational studies for a small series of azinesulfonamide analogs of aripiprazole, with tri- and tetra-methylene spacers and phenylpiperazine substituted with chlorine and methyl groups. Among azine fragments, the 7-quinolinyl fragment was the most favorable for interaction with 5-HT_1A_Rs. Moreover, the introduction of a chlorine atom or atoms into the phenyl ring significantly impacted the affinity for 5-HT_1A_ and 5-HT_7_Rs. Furthermore, conformational studies (X-ray analysis and 2D NMR experiments) of the polimethylene chain of LCAPs revealed that the bent conformation in a solid and in solutions is favorable. The above observations are compatible with the molecular modeling study performed for **2**–**6**.

Docking analysis of the tested compounds suggest that all LCAPs were consequently docked in their bent conformations, with synclinal C9–C10 torsion, and that they bind to 5-HT_1A_ and 5-HT_7_ receptors in a similar way. Our structural investigations organize knowledge about the conformational preferences of selected serotonin receptor ligands in different environments, and show that potentially bioactive conformations could be predicted by X-ray spectrometry and calculations using appropriate solvent simulated semi-empirical methods.

## Experimental

### Methods

Organic solvents (from Aldrich and Chempur) were of reagent grade and were used without purification.

Purity of the synthesized compounds was confirmed by TLC performed on Merck silica gel 60 F254 aluminium sheets (Merck, Darmstadt, Germany). Spots were detected by their absorption under UV light (k = 254 nm).

Analytical HPLC were run on a Waters Alliance HPLC instrument, equipped with a Chromolith SpeedROD column (4.6 × 50 mm). Standard conditions were eluent system A (water/0.1% TFA), system B (acetonitrile/0.1% TFA). A flow rate of 5 mL/min and a gradient of (0–100) % B over 3 min were used. Detection was performed on a PDA detector. Retention times (tR) are given in minutes.

NMR Spectra were recorded on Bruker Ascend 600 spectrometer operating at 600.22 and 125.12 MHz for ^1^H and ^13^C nuclei, respectively, in DMSO-d_6_ solution. Two-dimensional ^1^H-^1^H (COSY and NOESY) and ^1^H-^13^C (HSQC and HMBC) and NOE (ROESY) experiments were performed using standard Bruker software. J values are in hertz (Hz), and splitting patterns are designated as follows: s (singlet), brs (broad singlet) d (doublet), t (triplet), m (multiplet).

Mass spectrometry analyses—samples were prepared in acetonitrile/water (10/90 v/v) mixture. The LC/MS system consisted of a Waters Acquity UPLC, coupled to a Waters TQD mass spectrometer (electrospray ionization mode ESI-triple quadrupole (QqQ). All other analyses were carried out using a Acquity UPLC BEH C18, 50 × 2.1 mm reversed-phase column. A flow rate of 0.3 mL/min and a gradient of (5–95)% B over 5 min was used. Eluent A:water/0.1% HCO_2_H; eluent B: acetonitrile/0.1% HCO_2_H. Nitrogen was used for both nebulizing and drying gas. LC/MS data were obtained by scanning the first quadrupole in 0.5 s in a mass range from 100 to 700 m/z; 10 scans were summed up to produce the final spectrum.

Elemental analyses were found within ± 0.4% of the theoretical values. Melting points (mp) were determined with a Buchi apparatus and are uncorrected.

Column chromatography separations were carried out on column with Merck Kieselgel 60 or Aluminium oxide 90, neutral (70–230 mesh). Purification of compounds was performed on silica gel (irregular particles 40–63 lm, Merck Kieselgel 60).

### General procedure for the preparation of compounds **1**, **2**, and **4**

The starting 1-(4-aminobutyl)-4-phenylpiperazine (**9**), 1-(4-aminobutyl)-4-(3-chlorophenyl)piperazine (**10**), 1-(4-aminobutyl)-4-(2,3-dichlorophenyl)piperazine (**11**), 1-(4-aminobutyl)-4-(2,3-dimethylphenyl)piperazine (**12)**, and 1-(3-aminopropyl)-4-(2,3-dichlorophenyl)piperazine (**13**) were synthesized according to the Gabriel method. A mixture of the appropriate *N*-(ω-aminoalkyl)-phenylpiperazine (**9**–**13**) (1.0 mmol) in CH_2_Cl_2_ (7 mL) and DIEA (2.4 mmol) was cooled down (ice bath), and azinesulfonyl chloride **7** or **8** (1.2 mmol) was added at 0 °C in one portion. The reaction mixture was stirred for 6 h under cooling. Then, the solvent was evaporated and the sulfonamides were separated by column chromatography using SiO_2_ and a mixture of CH_2_Cl_2_/MeOH = 9/0.7 or 9/0.5, as an eluting system. Free bases were then converted into the hydrochloride salts by treatment of their solution in anhydrous ethanol with 1 M HCl in dioxane. The LC/MS of the identified compounds **1**–**6** exceeded purity of 98%.

### *N*-(4-(4-phenyl)piperazin-1-yl)butyl)isoquinoline-3-sulfonamide hydrochloride (**1**)

Yield 84%; m.p. 198–9 °C; ^1^H NMR δ: 1.43–1.46 (m, 2H, H-10), 1.68–1.72 (m, 2H, Hz, H-11), 2.94 (td, 2H, *J *= 6.6 Hz, *J *= 6.0 Hz, H-9), 3.05–3.49 (m, 10H, 10H, H-12, H-13, H-14, H-15 and H-16), 7.07–7.18 (m, 5H, Ph), 7.88 (dd, 1H, J = 8.4 Hz, J = 7.8 Hz, H-6), 7.93–7.99 (m, 2H, H-7 and –SO_2_NH–), 8.25–8.30 (m, 2H, H-5 and H-8), 8.52 (s, 1H, H-4), 9.49 (s, 1H, H-1), 10.95 (brs, 1H, –CH_2_NH^+^(CH_2_CH_2_)_2_N–); ^13^C NMR δ: 20.8 (C-11), 27.1 (C-10), 42.7 (C-9), 48.3 (C-14 and C-15), 51.8 (C-13 and C-16), 55.3 (C-12), 120.3 (C-4), 125.5 (Ph), 128.3 (Ph), 128.4 (C-5), 128.5 (C-3), 128.6 (Ph), 129.4 (C-8), 130.5 (C-6), 132.6 (C-7), 135.4 (C-4a), 139.3 (Ph), 151.5 (C-8a), 153.9 (C-1).

### *N*-(4-(4-(3-chlorophenyl)piperazin-1-yl)butyl)isoquinoline-3-sulfonamide hydrochloride (**2**)

Yield 89%; m.p. 246–7 °C; ^1^H NMR δ: 1.43–1.45 (m, 2H, H-10), 1.67–1.71 (m, 2H, H-11), 2.94 (td, 2H, *J *= 6.6 Hz, *J *= 6.0 Hz, H-9), 3.05–3.49 (m, 10H, H-12, H-13, H-14, H-15 and H-16), 6.87 (d, 1H, *J *= 7.2 Hz, Ph), 6.95 (d, 1H, *J *= 7.8 Hz, Ph), 7.07 (s, 1H, Ph), 7.25 (dd, 1H, *J *= 7.8 Hz, *J *= 7.2 Hz, Ph), 7.90 (dd, 1H, J = 8.4 Hz, J = 7.8 Hz, H-6), 7.93–7.98 (m, 2H, H-7 and –SO_2_NH–), 8.26–8.33 (m, 2H, H-5 and H-8), 8.53 (s, 1H, H-4), 9.48 (s, 1H, H-1), 10.06 (brs, 1H, –CH_2_NH^+^(CH_2_CH_2_)_2_N–); ^13^C NMR δ: 22.6 (C-11), 27.1 (C-10), 42.7 (C-9), 45.4 (C-14 and C-15), 50.9 (C-13 and C-16), 55.4 (C-12), 114.7 (Ph), 115.8 (Ph), 120.3 (C-4 and Ph), 128.4 (C-5), 128.5 (C-8), 129.4 (C-3), 130.5 (Ph), 131.1 (C-6), 132.6 (C-7), 134.4 (Ph), 135.4 (C-4a), 147.9 (Ph), 151.5 (C-8a), 154.0 (C-1).

### *N*-(4-(4-(2,3-dimethylphenyl)piperazin-1-yl)butyl)isoquinoline-3-sulfonamide hydrochloride (**4**)

Yield 86%; m.p. 215–6 °C; ^1^H NMR δ: 1.44–1.48 (m, 2H, H-10), 1.71–1.75 (m, 2H, *J *= 6.6 Hz, H-11), 2.15 (s, 3H, –CH_3_), 2.21 (s, 3H, –CH_3_), 2.93 (td, 2H, *J *= 6.6 Hz, *J *= 6.0 Hz, H-9), 3.08–3.46 (m, 10H, H-12, H-13, H-14, H-15 and H-16), 6.87–6.91 (m, 2H, Ph), 7.04–7.09 (m, 1H, Ph), 7.86–7.99 (m, 3H, H-6, H-7 and –SO_2_NH), 8.25–8.31 (m, 2H, H-5 and H-8), 8.53 (s, 1H, H-4), 9.49 (s, 1H, H-1), 10.93 (brs, 1H, –CH_2_NH^+^(CH_2_CH_2_)_2_N–); ^13^C NMR δ: 14.1 (–CH_3_), 20.7 (–CH_3_), 20.8 (C-11), 27.1 (C-10), 42.7 (C-9), 48.9 (C-14 and C-15), 51.7 (C-13 and C-16), 55.4 (C-12), 116.9 (Ph), 120.3 (C-4), 125.9 (Ph), 126.3 (Ph), 128.4 (C-5), 128.5 (C-3), 129.4 (C-8), 130.5 (C-6), 131.0 (Ph), 132.6 (C-7), 135.4 (C-4a), 138.1 (Ph), 150.3 (Ph), 151.5 (C-8a), 153.9 (C-1).

### X-ray crystal structures determination

Crystals of **2**–**6** were obtained by slow evaporation of the solvent under ambient conditions from ethanol: water mixture in ratio of 5:1.

X-ray diffraction data were collected using SuperNova diffractometer with Cu Kα radiation (λ = 1.54184 Å) for crystal **2** and with Mo Kα radiation (λ = 0.71073 Å) for crystals **3**–**6**, with the CrysAlisPro software [34]. Data were processed with the same program. Experiments were performed at 100 K excepting crystal of **4**, which was measured at room temperature. The phase problem was solved by direct methods with SHELXS-97 [35]. The model parameters were refined by full-matrix least-squares on F^2^ using SHELXL-2014/7 [35]. All non-hydrogen atoms were refined anisotropically. Hydrogen atoms were introduced to all structures by appropriate rigid body constraints (AFIX 23, AFIX 43 or AFIX 137) with temperature factors U_iso_(H) equal to 1.2Ueq(C) for aromatic and methylene hydrogen atoms or 1.5Ueq(C) for methyl hydrogen atoms. Hydrogen atoms which take part in the hydrogen bonds were located in the calculated positions and then freely refined. Due to severely disordered solvent in crystal of **2**, the SQUEEZE program was used [36]. All crystallographic data for presented structures are shown in Additional file 1: Table S5. The figures showing asymmetric units were made with Jmol [37]. The figure presenting structural motives were made with Mercury [38].

### In vitro evaluation

#### Cell culture and preparation of cell membranes for radioligand binding assays

HEK293 cells with stable expression of human 5-HT_1A_, 5-HT_6_, 5-HT_7b_ and D_2L_ receptors (prepared with the use of Lipofectamine 2000) were maintained at 37 °C in a humidified atmosphere with 5% CO_2_ and grown in Dulbecco’s Modifier Eagle Medium containing 10% dialyzed fetal bovine serum and 500 µg/ml G418 sulfate. For membrane preparation, cells were subcultured in 150 cm^2^ flasks, grown to 90% confluence, washed twice with prewarmed to 37 °C phosphate buffered saline (PBS) and pelleted by centrifugation (200*g*) in PBS containing 0.1 mM EDTA and 1 mM dithiothreitol. Prior to membrane preparation, pellets were stored at − 80 °C.

#### Radioligand binding assays

Cell pellets were thawed and homogenized in 10 volumes of assay buffer using an Ultra Turrax tissue homogenizer and centrifuged twice at 35,000*g* for 15 min at 4 °C, with incubation for 15 min at 37 °C in between. The composition of the assay buffers was as follows: for 5-HT_1A_R: 50 mM Tris HCl, 0.1 mM EDTA, 4 mM MgCl_2_, 10 µM pargyline and 0.1% ascorbate; for 5-HT_6_R: 50 mM Tris HCl, 0.5 mM EDTA and 4 mM MgCl_2_, for 5-HT_7b_R: 50 mM Tris HCl, 4 mM MgCl_2_, 10 µM pargyline and 0.1% ascorbate; for dopamine D_2L_R: 50 mM Tris HCl, 1 mM EDTA, 4 mM MgCl_2_, 120 mM NaCl, 5 mM KCl, 1.5 mM CaCl_2_ and 0.1% ascorbate. All assays were incubated in a total volume of 200 µl in 96-well microtitre plates for 1 h at 37 °C, except 5-HT_1A_R which were incubated at room temperature. The process of equilibration was terminated by rapid filtration through Unifilter plates with a FilterMate Unifilter 96-Harvester (PerkinElmer). The radioactivity bound to the filters was quantified on a Microbeta TopCount instrument (PerkinElmer, USA). For competitive inhibition studies the assay samples contained as radioligands (PerkinElmer, USA): 2.5 nM [^3^H]-8-OH-DPAT (135.2 Ci/mmol) for 5-HT_1A_R; 2 nM [^3^H]-LSD (83.6 Ci/mmol) for 5-HT_6_R; 0.8 nM [^3^H]-5-CT (39.2 Ci/mmol) for 5-HT_7_R or 2.5 nM [^3^H]-raclopride (76.0 Ci/mmol) for D_2L_R. Non-specific binding was defined with 10 µM of 5-HT in 5-HT_1A_R and 5-HT_7_R binding experiments, whereas 10 µM of methiothepine or 10 µM of haloperidol were used in 5-HT_6_R and D_2L_ assays, respectively. Each compound was tested in triplicate at 7 concentrations (10^−10^–10^−4^ M). The inhibition constants (*K*_i_) were calculated from the *Cheng*–*Prusoff* equation [39]. Results were expressed as means of at least two separate experiments.

### Computational details

#### Geometry optimization

Ab initio calculations of the studied azinesulfonamides, using crystallographic data as starting point, were carried out with the Gaussian 16 (revision A.03) computer code [40] at the density functional theory (DFT, Becke3LYP [41]) level of theory using the 6–311 + G(d,p) basis sets. The conformational behavior of these systems in water was examined using the CPCM solvation method [42, 43].

#### Molecular docking

3-Dimensional structures of the ligands were prepared using LigPrep v3.6 [44], and the appropriate ionization states at pH 7.4 ± 1.0 were assigned using Epik v3.4 [45]. The Protein Preparation Wizard was used to assign the bond orders, appropriate amino acid ionization states and to check for steric clashes. The receptor grid was generated (OPLS3 force field [46]) by centering the grid box with a size of 12 Å on Asp3.32 residue. Docking was performed by quantum-polarized ligand docking (QPLD) procedure [47] involves the QM-derived ligand atomic charges in the protein environment at the B3PW91/cc-pVTZ level. Only ten best poses per ligand returned by the procedure were considered.

#### Binding free energy calculations

MM/GBSA (Generalized-Born/Surface Area) was used to calculate the binding free energy based on the ligand–receptor complexes generated by the QPLD procedure. The ligand poses were minimized using the local optimization feature in Prime, the flexible residue distance was set to 4.0 Å from a ligand pose, and the ligand charges obtained in the QPLD stage were used. The energies of complexes were calculated with the OPLS3 force field and Generalized-Born/Surface Area continuum solvent model. To assess the influence of a given substituent on the binding, the ∆∆G was calculated as a difference between binding free energy (∆G) of unsubstituted at phenyl ring sulfonamide 1 and investigated analogs 3, 4, and 5.

#### Plotting interaction spheres for halogen bonding

To visualize (plotting interaction spheres) the possible contribution of halogen bonding to ligand–receptor complexes, the halogen bonding web server was used (access Oct 01, 2017, http://www.halogenbonding.com/).

## Additional file

**Additional file 1: Figure S1.** Histograms of population of different arylpiperazine salts conformations. **Figure S2**. Low-energy conformation of the studied compounds in aqueous medium. **Table S1**. Conformation of arylpiperazine derivatives with polymethylene spacer (H-N+(CH2)nN-Y) in the crystal state (n=3 and 4). **Table S2**. Conformation of phenyl ring and piperazine moiety in arylpiperazine derivatives with polymethylene spacer [H-N+-(CH2)n-X] in the crystal state (n=2-4). **Table S3**. Strong and weak hydrogen bonds geometry for structures 2-6 [Å and °]. **Table S4**. Optimized dihedral angles [°] of the studied compounds. **Table S5**. Crystal data and structure refinement.
