# Supplementary material for: Structural determinants influencing halogen bonding: a case study on azinesulfonamide analogs of aripiprazole as 5-HT1A, 5-HT7, and D2 receptor ligands
Source: Chem Cent J. 2018 May 11;12:55. doi: 10.1186/s13065-018-0422-5 (PMC5945563; doi:10.1186/s13065-018-0422-5)
Supplement: Supplementary file 1 — Additional file 1: Figure S1. Histograms of population of different arylpiperazine salts conformations. Figure S2. Low-energy conformation of the studied compounds in aqueous medium. Table S1. Conformation of arylpiperazine derivatives with polymethylene spacer (H-N+(CH2)nN-Y) in the crystal state (n=3 and 4). Table S2. Conformation of phenyl ring and piperazine moiety in arylpiperazine derivatives with polymethylene spacer [H-N+-(CH2)n-X] in the crystal state (n=2-4). Table S3. Strong and weak hydrogen bonds geometry for structures 2-6 [Å and °]. Table S4. Optimized dihedral angles [°] of the studied compounds. Table S5. Crystal data and structure refinement. [file 13065_2018_422_MOESM1_ESM.docx]

**Structural determinants influencing halogen bonding: a case study on azinesulfonamide analogs of aripiprazole as 5-HT_1A_, 5-HT_7_, and D_2_ receptor ligands**

Krzysztof Marciniec^a^*, Rafał Kurczab^b^, Maria Książek^c^, Ewa Bębenek^a^, Elwira Chrobak^a^, Grzegorz Satała^b^, Andrzej J. Bojarski^b^, Joachim Kusz^c^, Paweł Zajdel^d^

**Additional file**

**Table S1**. Conformation of arylpiperazine derivatives with polymethylene spacer (H-N^+^(CH_2_)_n_N-Y) in the crystal state^a^ (n=3 and 4).

| Category  Structure | *N*^+^...*N*-*Y* [Å] | τ_1_ [º] | τ_1’_ [º]^b^ | τ_2_ [º] | τ_3_ [º] | τ_4_ [º] | τ_5_ [º]^c^ |
| --- | --- | --- | --- | --- | --- | --- | --- |
| n=3 | | | | | | | |
| **6** | 4.39 | -102.7 | - | -68.3 | 179.7 | -55.1 | - |
| IWEJAV | 5.04 | -61.8 | 59.6 | 168.43 | 162.2 | -50.9 | - |
| EZEYUE | 4.98 | -73.3 | 101.9 | 168.8 | 150.5 | 42.2 | - |
| AJUZUA | 5.09 | -57.0 | 63.5 | -179.2 | 176.6 | 172.7 | - |
| AJUZUA01 | 5.09 | -56.9 | 63.4 | -179.3 | 176.5 | 171.9 | - |
| n=4 | | | | | | | |
| **2** | 5.47 | -113.7 | - | -69.0 | 179.4 | -174.8 | 53.7 |
| **3** | 5.48 | -156.3 | - | 74.4 | -170.5 | -176.5 | -49.5 |
| **4** | 5.48 | -155.7 | - | 73.9 | -171.4 | -176.2 | -50.5 |
| **5** | 5.00 | -119.3 | - | -65.1 | -72.7 | 178.3 | -53.7 |
| MECGIM | 5.50 | 108.3 | - | -69.0 | 178.9 | 175.9 | 162.7 |
| PECHIQ | 4.95 | 78.8 | - | 68.3 | 178.1 | 77.1 | -60.9 |
| KATCIT | 6.25 | 127.9 | - | 164.8 | 173.8 | 177.8 | 58.3 |
| OFAZEA | 6.29 | 108.9 | -80.0 | 172.9 | -176.9 | -178.3 | 55.8 |
| SOBVOW | 6.22 | 104.5 | -79.8 | -166.2 | 173.0 | -164.2 | 58.4 |
| SOBVUC | 6.25 | -113.1 | 84.3 | 179.2 | 171.2 | 175.2 | 51.7 |
| SOBWAJ | 6.22 | -104.8 | 82.9 | 166.5 | -172.7 | 167.5 | -55.9 |
| SOBWEN | 6.29 | -113.0 | 84.8 | 164.4 | 171.5 | 175.6 | 36.8 |
| OFAZOK | 5.79 | -91.6 | 92.9 | -168.7 | -72.4 | 166.2 | 67.7 |
| OFAZIE | 5.90 | -116.6 | 62.8 | -162.7 | -75.7 | 172.8 | 47.6 |

^a^ Selected CSD crystals satisfying following conditions: (i) - no substitution in the alkyl chain; (ii) – protonated piperazine nitrogen atom (as it is expected to be while interacting with the receptors),

^b^ due to planarity of terminal group both τ_1_ and τ_1’_ are listed.

(τ_5_) defined as C11-C12-N^+^-H

**Table S2**. Conformation of phenyl ring and piperazine moiety in arylpiperazine derivatives with polymethylene spacer [H-N^+^-(CH_2_)_n_-X] in the crystal state (n=2-4).

| Category  Structure | τ_6_ [^o^] | Dihedral angle piperazine/aryl ф [^o^] |
| --- | --- | --- |
| **2** | -147.5 | 20.6 |
| **3** | 159.2 | 47.1 |
| **4** | 158.8 | 48.0 |
| **5** | -143.8 | 62.8 |
| **6** | 154.6 | 51.5 |
| AJUZUA | 162.6 | 35.8 |
| AJUZUA01 | 162.6 | 43.9 |
| AKABAP | 163.3 | 45.4 |
| AKABAP01 | 163.3 | 45.4 |
| AKIMIQ | 17.2 | 19.2 |
| COLDAK | 119.6 | 86.6 |
| CPTAZP | 40.1 | 26.2 |
| DAYQAW | 156.7 | 48.9 |
| EDUWIK | 31.0 | 14.0 |
| EZEYUE | 150.3 | 56.5 |
| EZEZEP | 162.7 | 57.7 |
| EZEZIT | 26.4 | 19.0 |
| FAXLUN | 66.6 | 45.8 |
| FOPYUE | 174.2 | 34.1 |
| HAXQUU | 115.6 | 44.4 |
| HIJCOS | 164.4 | 41.5 |
| IWEJAV | 170.4 | 36.4 |
| KATCIT | 69.1 | 44.9 |
| KIYFUT | 17.8 | 15.3 |
| LERJUP | 74.3 | 48.6 |
| MECGIM | 157.6 | 49.1 |
| MUXZEK | 65.8 | 86.7 |
| OFAZEA | 165.5 | 40.7 |
| OFAZIE | 14.3 | 19.9 |
| OFAZOK | 27.4 | 6.7 |
| OXYPEB10 | 43.0 | 23.0 |
| PECHIQ | 166.2 | 39.0 |
| PEZNEP | 73.0 | 47.3 |
| PEZNIT | 155.4 | 50.6 |
| PEZNOZ | 162.1 | 44.0 |
| PEZNUF | 164.4 | 43.0 |
| SOBVOW | 52.3 | 33.8 |
| SOBVUC | 30.0 | 22.3 |
| SOBWAJ | 167.3 | 39.7 |
| SOBWEN | 43.1 | 22.4 |
| WANCET | 49.4 | 31.3 |

**Figure S1**. Histograms of population of different arylpiperazine salts conformations.

**Table S3**. Strong and weak hydrogen bonds geometry for structures **2**-**6** [Å and ˚].

| **Compound** | **D-H^…^A** | **d(D-H)** | **d(H^…^A)** | **d(D^…^A)** | **<(DHA)** |
| --- | --- | --- | --- | --- | --- |
| **2** | N(14)-H(14N)^…^Cl(1) | 1.00 | 2.08 | 3.066(7) | 170.0 |
|  | C(11)-H(11)^…^Cl(2)^i^ | 0.95 | 2.75 | 3.644(11) | 156.4 |
|  | C(110)-H(11A)^…^O(11)^ii^ | 0.99 | 2.58 | 3.358(13) | 135.2 |
|  | C(112)-H(11E)^…^Cl(1)^ii^ | 0.99 | 2.95 | 3.810(9) | 146.3 |
|  | C(112)-H(11F)^…^Cl(1)^iii^ | 0.99 | 2.71 | 3.680(10) | 165.9 |
|  | C(113)-H(11G)^…^Cl(1)^ii^ | 0.99 | 2.97 | 3.831(9) | 146.3 |
|  | C(115)-H(11K)^…^O(12)^iii^ | 0.99 | 2.47 | 3.223(11) | 132.2 |
|  | C(116)-H(11N)^…^Cl(1)^ii^ | 0.99 | 2.81 | 3.709(8) | 152.0 |
|  | C(21)-H(21)^…^Cl(1) | 0.95 | 2.99 | 3.90(10) | 161.5 |
|  | C(210)-H(21B)^…^O(21)^ii^ | 0.99 | 2.64 | 3.441(10) | 137.7 |
|  | C(214)-H(21I)^…^O(22)^iv^ | 0.99 | 2.62 | 3.383(13) | 133.5 |
|  | C(215)-H(21K)^…^Cl(2) | 0.99 | 2.88 | 3.562(13) | 126.9 |
|  | C(218)-H(218)^…^O(22)^iv^ | 0.95 | 2.51 | 3.454(13) | 171.0 |
| **3** | N(3)-H(3N)^…^Cl(3) | 0.81(4) | 2.45(4) | 3.239(3) | 167(4) |
|  | N(4)-H(4N)^…^Cl(3)^v^ | 0.95(4) | 2.11(4) | 3.050(3) | 174(3) |
| **4** | N(3)-H(99)^…^Cl(1) | 0.94(2) | 2.13(3) | 3.067(2) | 175(2) |
|  | N(4)-H(2)^…^Cl(1)^vi^ | 0.85(3) | 2.43(3) | 3.251(2) | 164(2) |
|  | C(13)-H(13B)···Cl(1)^vii^ | 0.99(3) | 2.76(3) | 3.684(3) | 155.3(19) |
|  | C(15)-H(15B)···Cl(1)^viii^ | 0.96(3) | 2.94(3) | 3.602(3) | 127.2(19) |
|  | C(16)-H(16A)···Cl(1)^vii^ | 0.93(3) | 2.90(3) | 3.738(3) | 150(2) |
|  | C(16)-H(16B)···O(2)^vii^ | 0.98(3) | 2.58(3) | 3.532(3) | 166(2) |
| **5** | N(3)-H(3N)^…^O(1W) | 0.79(4) | 2.00(4) | 2.789(3) | 176(3) |
|  | N(4)-H(4N)^…^Cl(3) | 0.88(3) | 2.15(3) | 3.015(2) | 168(2) |
|  | O(1W)-H(1W)^…^O(2)^ii^ | 0.83(4) | 2.08(4) | 2.903(3) | 170(4) |
|  | O(1W)-H(2W)^…^Cl(3) | 0.91(5) | 2.17(5) | 3.076(2) | 173(4) |
| **6** | N(2)-H(2N)^…^Cl(3) | 0.84(5) | 2.38(5) | 3.133(2) | 150(4) |
|  | N(3)-H(3N)^…^Cl(3)^ix^ | 0.94(4) | 2.08(4) | 3.17(3) | 174(4) |

Symmetry transformations used to generate equivalent atoms: i: -x+1,-y+1,-z+2; ii: x-1,y,z; iii: -x+1,-y+1,-z+1; iv: -x+1,-y,-z+2; v: -x,y-1/2,-z+3/2; vi: -x+1,y+1/2,-z+3/2; vii: x,-y+1/2,z-1/2; viii: -x+1,-y,-z+1; ix: x,y+1,z

**Table S4**. Optimized^a^ dihedral angles [º] of the studied compounds.

| **Compound** | | **2** | **3** | **4** | **5** | **6** |
| --- | --- | --- | --- | --- | --- | --- |
| τ_1_ [º] | X-ray | -113.7 | -156.3 | -155.7 | -119.3 | -102.7 |
|  | DFT | -85.1 | -109.8 | -110.0 | -64.6 | -75.8 |
|  | DFT-CPCM | -83.3 | -151.7 | -152.5 | -94.8 | -89.8 |
|  | Docking 5-HT_7_^b^ | -105.6 | -144.6 | -143.6 | -102.4 | -96.1 |
|  | Docking 5-HT_1A_ | -95.5 | -128.7 | -125.6 |  | -71.5 |
| τ_2_ [º] | X-ray | -69.0 | 74.4 | 73.9 | -65.1 | -68.3 |
|  | DFT | -53.7 | 61.3 | 61.9 | -40.3 | -45.7 |
|  | DFT-CPCM | -61.9 | 64.7 | 64.9 | -68.3 | -62.0 |
|  | Docking 5-HT_7_ | -62.6 | 70.4 | 69.4 | -67.4 | -65.6 |
|  | Docking 5-HT_1A_ | -72.9 | 68.4 | 63.7 | -78.6 | -93.6 |
| τ_3_ [º] | X-ray | 179.4 | -170.5 | -171.4 | -72.7 | 179.7 |
|  | DFT | 166.2 | -179.6 | -179.0 | -41.0 | 96.9 |
|  | DFT-CPCM | -176.9 | 179.1 | 178.8 | 179.9 | 169.4 |
|  | Docking 5-HT_7_ | -170.6 | -169.4 | -161.7 | -42.9 | 167.5 |
|  | Docking 5-HT_1A_ | -168.4 | -165.8 | -161.5 | -61.1 | 165.1 |
| τ_4_ [º] | X-ray | -174.8 | -176.5 | -176.2 | 178.3 | -55.1 |
|  | DFT | -157.1 | 166.0 | 166.6 | 86.9 | -152.2 |
|  | DFT-CPCM | -174.3 | 172.2 | 173.0 | 173.2 | -170.2 |
|  | Docking 5-HT_7_ | -175.3 | -173.9 | -177.8 | -170.6 | -59.1 |
|  | Docking 5-HT_1A_ | -174.5 | -162.6 | -161.1 | -164.9 | -75.7 |
| τ_5_ [º] | X-ray | 53.7 | -49.5 | -50.5 | -53.7 | - |
|  | DFT | 170.1 | -172.2 | -171.2 | -163.2 | - |
|  | DFT-CPCM | 168.6 | -170.2 | -169.5 |  | - |
|  | Docking 5-HT_7_ | 59.1 | -57.9 | -52.8 | -53.2 | - |
|  | Dockin 5-HT_1A_ | -72.2 | -80.9 | -75.8 | -62.5 | - |
| τ_6_ [ º] | X-ray | -147.5 | 159.2 | 158.8 | -143.8 | 154.6 |
|  | DFT | -117.6 | 148.9 | 146.9 | -149.2 | 149.3 |
|  | DFT-CPCM | -131.5 | 152.5 | 153.0 | -153.0 | 152.6 |
|  | Docking 5-HT_7_ | -152.7 | 138.1 | 125.9 | 122.7 | 116.5 |
|  | Dockin 5-HT_1A_ | -151.8 | 154.2 | 155.5 | -144.8 | 116.1 |

^a^ B3LYP/6-311+G(d,p)

^b^ the average dihedral value calculated for the best binding poses found in all considered receptors


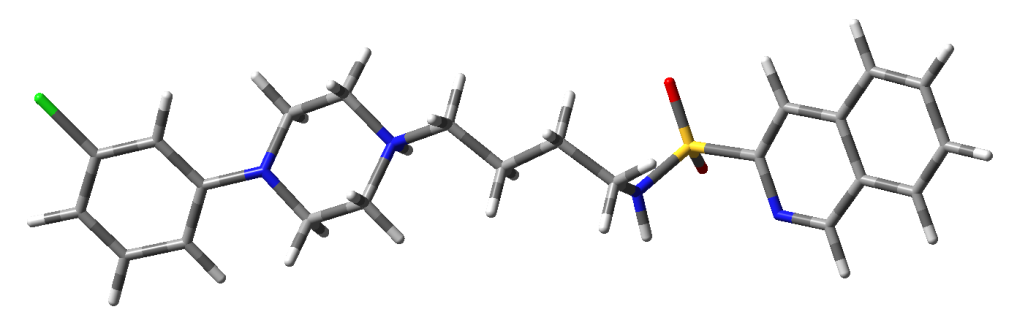

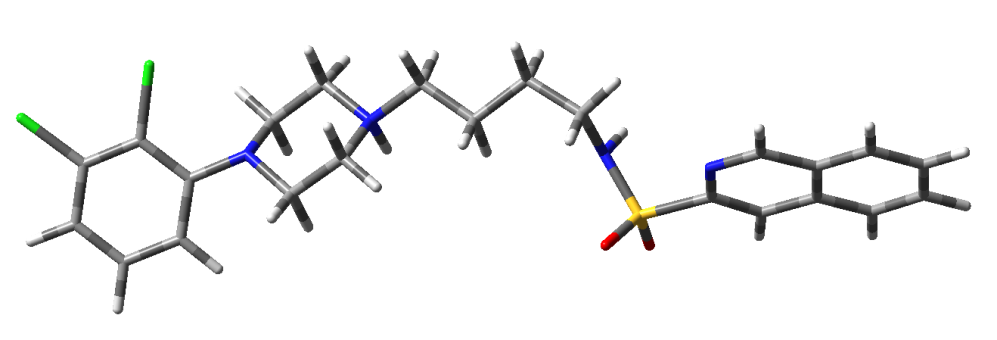


**2 3**


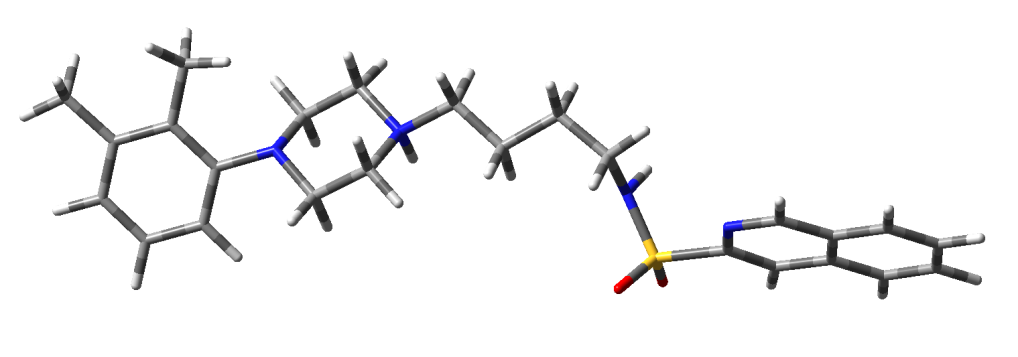

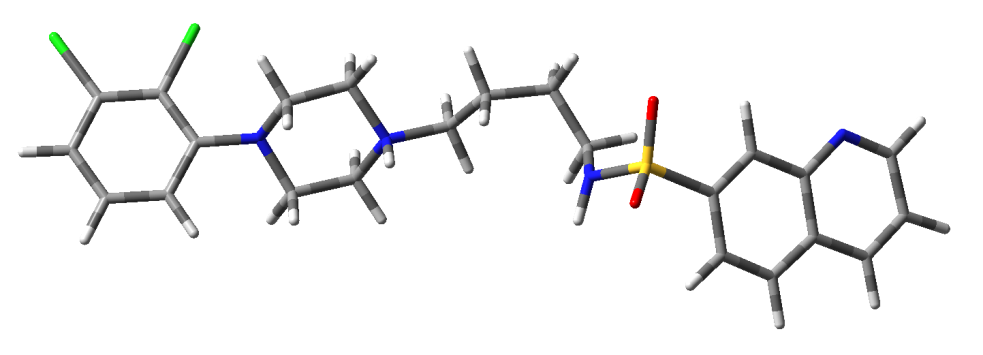


**4 5**

**
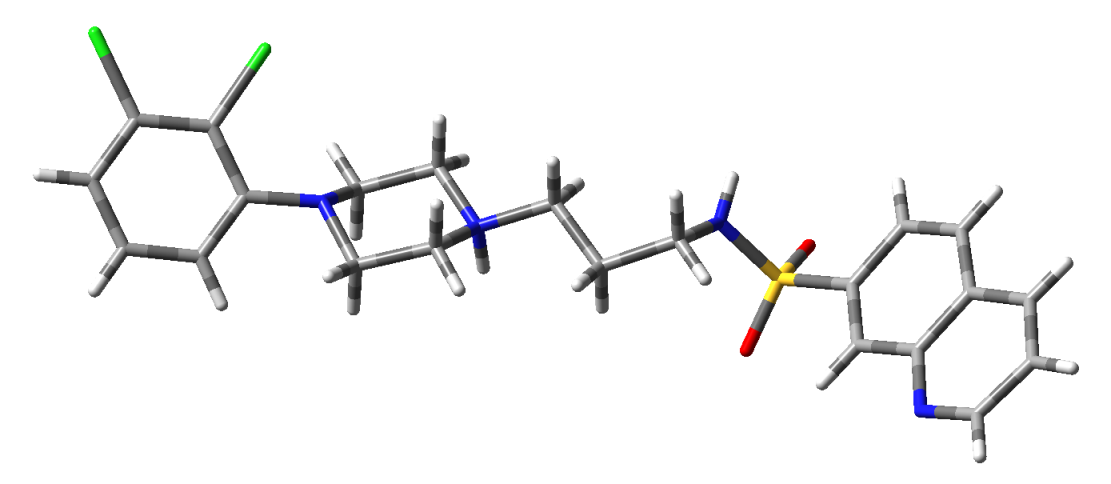
**

**6**

**Figure S2**. Low-energy conformation of the studied compounds in aqueous medium

**Table S5**. Crystal data and structure refinement

|  | **2** | **3** | **4** | **5** | **6** |
| --- | --- | --- | --- | --- | --- |
| Molecular formula | [C_23_H_28_ClN_4_O_2_S]^+^Cl^-^ | [C_23_H_27_Cl_2_N_4_O_2_S]^+^Cl^-^ | [C_25_H_33_N_4_O_2_S]^+^Cl^-^ | [C_23_H_27_Cl_2_N_4_O_2_S]^+^Cl^-^ · H_2_O | [C_22_H_25_Cl_2_N_4_O_2_S]^+^Cl^-^ |
| Formula weight | 495.45 | 529.90 | 489.06 | 547.91 | 515.87 |
| Crystal system | triclinic | monoclinic | monoclinic | monoclinic | monoclinic |
| Space group | P-1 | P2_1_/c | P2_1_/c | Pc | P2_1_/c |
| Unit cell dimentions [Å] | *a* = 7.0093(6)  *b* = 20.368(3)  *c* = 20.918(3)  α = 63.049(13)˚  β = 81.543(9)˚  γ = 85.518(8)˚ | *a* = 15.2389(3)  *b* = 13.5028(3)  *c* = 11.8726(2)  α = 90.00˚  β = 94.117(2)˚  γ = 90.00˚ | *a* = 15.1703(6)  *b* = 13.7582(5)  *c* = 11.8775(4)  α = 90.00˚  β = 93.327(3)˚  γ = 90.00˚ | *a* = 6.9116(2)  *b* = 29.3055(4)  *c* = 6.9926(2)  α = 90.00˚  β = 118.088(3)˚  γ = 90.00˚ | *a* = 25.9210(3)  *b* = 6.8213(1)  *c* = 13.3821(2)  α = 90.00˚  β = 94.647(1)˚  γ = 90.00˚ |
| Volume [Å^3^] | 2632.8(6) | 2436.69(8) | 2474.85(16) | 1249.53(5) | 2358.38(6) |
| *Z* | 4 | 4 | 4 | 2 | 4 |
| D_calc_ [g/cm^3^] | 1.250 | 1.444 | 1.313 | 1.456 | 1.453 |
| Absorption coefficient  [mm^-1^] | 3.166 | 0.491 | 0.269 | 0.484 | 0.505 |
| *F*(000) | 1040 | 1104 | 1040 | 572 | 1072 |
| Crystal size [mm^3^] | 0.12 x 0.02 x 0.02 | 0.10 x 0.10 x 0.01 | 0.08 x 0.06 x 0.02 | 0.13 x 0.10 x 0.02 | 0.25 x 0.17 x 0.02 |
| θ range [˚] | 2.39-58.93 | 2.29-25.06 | 2.27-25.68 | 2.08-25.06 | 2.36-25.06 |
| Diffractometer | SuperNova diffractometer (Agilent Technologies) with Atlas detector | | | | |
| Index ranges | -6 ≤ h ≤ 7  -21 ≤ k ≤ 22  -23 ≤ l ≤ 23 | -18 ≤ h ≤ 17  -16 ≤ k ≤ 14  -14 ≤ l ≤ 14 | -18 ≤ h ≤ 18  -16 ≤ k ≤ 16  -14 ≤ l ≤ 13 | -8 ≤ h ≤ 8  -34 ≤ k ≤ 33  -8 ≤ l ≤ 8 | -30 ≤ h ≤ 30  -6 ≤ k ≤ 8  -15 ≤ l ≤ 15 |
| Reflection collected | 14316 | 32661 | 19598 | 17121 | 31346 |
| Independent reflns | 6966 | 4316 | 4707 | 3358 | 4169 |
| Data/parameters | 6966/577 | 4316/304 | 4707/325 | 3358/321 | 4169/295 |
| Refinement method | Full-matrix least-squares on F^2^ | | | | |
| S (on F^2^) | 1.244 | 1.283 | 1.063 | 0.996 | 1.304 |
| R indices [I>2σ(I)] | R1 = 0.1205,  wR2 = 0.3321 | R1 = 0.0446,  wR2 = 0.1134 | R1 = 0.0480,  wR2 = 0.1180 | R1 = 0.0193,  wR2 = 0.0530 | R1 = 0.0501,  wR2 = 0.1208 |
| R indices (all data) | R1 = 0.1575,  wR2 = 0.3622 | R1 = 0.0475,  wR2 = 0.1145 | R1 = 0.0720,  wR2 = 0.1361 | R1 = 0.0194,  wR2 = 0.0531 | R1 = 0.0509,  wR2 = 0.1211 |
| Δρ min/max [eÅ^3^] | -0.758, 0.882 | -0.405, 0.471 | -0.286, 0.339 | -0.156, 0.219 | -0.403, 0.365 |
